# Equivalence trial of proposed denosumab biosimilar GP2411 and reference denosumab in postmenopausal osteoporosis: the ROSALIA study

**DOI:** 10.1093/jbmr/zjae016

**Published:** 2024-01-29

**Authors:** Sławomir Jeka, Eva Dokoupilová, Alan Kivitz, Paweł Żuchowski, Barbara Vogg, Natalia Krivtsova, Susmit Sekhar, Samik Banerjee, Arnd Schwebig, Johann Poetzl, Jean-Jacques Body, Richard Eastell

**Affiliations:** Clinic and Department of Rheumatology and Systemic Diseases of Connective Tissue, University Hospital No. 2. Bydgoszcz, CM UMK, 85-168 Bydgoszcz, KP, Poland; Medical Plus, s.r.o., 68601 Uherske Hradiste, ZL, Czech Republic; Masaryk University, Faculty of Pharmacy, Department of Pharmaceutical Technology, 612 00 Brno, JM, Czech Republic; Altoona Center for Clinical Research, Duncansville, PA 16635, United States; Clinic and Department of Rheumatology and Systemic Diseases of Connective Tissue, University Hospital No. 2. Bydgoszcz, CM UMK, 85-168 Bydgoszcz, KP, Poland; Clinical Development Biopharmaceuticals, Hexal AG, 83607 Holzkirchen, BY, Germany; Clinical Development Biopharmaceuticals, Hexal AG, 83607 Holzkirchen, BY, Germany; Clinical Development Biopharmaceuticals, Hexal AG, 83607 Holzkirchen, BY, Germany; Clinical Development Biopharmaceuticals, Hexal AG, 83607 Holzkirchen, BY, Germany; Clinical Development Biopharmaceuticals, Hexal AG, 83607 Holzkirchen, BY, Germany; Clinical Development Biopharmaceuticals, Hexal AG, 83607 Holzkirchen, BY, Germany; Department of Medicine, University Hospital Brugmann, Université Libre de Bruxelles (ULB), 1020 Brussels, BE, Belgium; Division of Clinical Medicine, University of Sheffield, Sheffield S10 2TN, SYK, United Kingdom

**Keywords:** diseases and disorders of/related to bone: osteoporosis, clinical trials, bone modeling and remodeling: biochemical markers of bone turnover

## Abstract

Denosumab is a monoclonal antibody used to reduce risk of fractures in osteoporosis. ROSALIA was a multicenter, double-blind, randomized, integrated phase I/phase III study comparing the efficacy, pharmacokinetics (PK), pharmacodynamics (PD), immunogenicity, and safety of proposed biosimilar denosumab GP2411 with reference denosumab (REF-DMAb) (Prolia®; Amgen). Postmenopausal women with osteoporosis were randomized 1:1 to 2 60-mg doses of GP2411 or REF-DMAb, one at study start and one at week 26. At week 52, the REF-DMAb group was re-randomized 1:1 to a third dose of REF-DMAb or switch to GP2411. The primary efficacy endpoint was percentage change from baseline (%CfB) in LS-BMD at week 52. Secondary efficacy endpoints were %CfB in LS-BMD, FN-BMD, and TH-BMD at weeks 26 and 78 (and week 52 for FN-BMD and TH-BMD). Primary PK and PD endpoints were the area under the serum concentration–time curve extrapolated to infinity and maximum drug serum concentration at week 26, and the area under the effect–time curve of the %CfB in serum CTX at week 26. Secondary PK and PD endpoints included drug serum concentrations and %CfB in serum CTX and P1NP during the study period. Similar efficacy was demonstrated at week 52, with 95% CIs of the difference in %CfB in LS-BMD between treatment groups fully contained within prespecified equivalence margins. Similarity in PK and PD was demonstrated at week 26. Immunogenicity was similar between groups and was not impacted by treatment switch. The rate of new vertebral fractures was comparable. Treatment-emergent adverse events were comparable between groups (63.6% [GP2411/GP2411]; 76.0% [REF-DMAb/REF-DMAb]; 76.6% [REF-DMAb/GP2411]). In conclusion, ROSALIA showed similar efficacy, PK and PD, and comparable safety and immunogenicity of GP2411 to REF-DMAb in postmenopausal osteoporosis.

## Lay Summary

Denosumab is a biologic treatment that stops bone breakdown. This clinical trial evaluated how similar GP2411 (a denosumab biosimilar in development) is compared with European-approved reference denosumab in women with post-menopausal osteoporosis. Biosimilars are highly similar to the original treatment (‘reference denosumab’) and may have a lower price. 263 patients were randomly assigned to receive GP2411 and 264 to reference denosumab. Treatment was given at the study beginning, at Week 26 and at Week 52. 124 patients were re-assigned at Week 52 to test the effect of changing from reference denosumab to GP2411. The study showed similarity in how the body interacts with the treatments, what effects the treatment has (both measured over 26 weeks), and bone mineral density (measured over 78 weeks). Antibody responses to GP2411 were detected in similar proportions of patients on each treatment. Reported adverse events were similar between treatments before Week 52, and from Week 52 to 78, and <5% of patients experienced serious adverse events. A change of treatment from reference denosumab to GP2411 did not affect outcomes. These results showed similarity between GP2411 and reference denosumab in this population. In future, GP2411 may enable more patients to benefit from denosumab.

## Introduction

Denosumab is an engineered humanized IgG2 monoclonal antibody that is effective for reducing fracture rates in patients with osteoporosis, reducing hormonal treatment-associated bone loss, and/or reducing skeletal-related events in the oncologic setting.^1-4^ Denosumab binds to the RANKL, which plays a key role in osteoclast-mediated bone resorption. By binding to RANKL, it prevents activation of RANK and inhibits osteoclast formation, function, and survival, thus inhibiting bone resorption.^1-4^ Denosumab is a well-established therapy that provides a sustained reduction in the risk of osteoporotic fractures.^5^

The price of reference denosumab (REF-DMAb) product is high compared with widely used therapies for osteoporosis.^6^^,^^7^ A denosumab biosimilar may be valuable, since biosimilars are typically cheaper to produce than the reference product, which can lead to cost savings and improved access to treatment, benefitting patients and healthcare systems.^8^

Biosimilar medicines are approved by the US Food and Drug Administration (FDA) and European Medicines Agency (EMA) on the basis of a stepwise approach that shows that the biosimilar matches the reference medicine in analytical and functional characterization studies; clinical pharmacology studies of pharmacokinetics (PK) and pharmacodynamics (PD); and confirmatory clinical studies evaluating efficacy, safety, and immunogenicity.^9-11^ Sandoz is developing GP2411, a biosimilar to Amgen’s denosumab (reference product). At the time of manuscript writing, licensing applications were under evaluation by health authorities. The similarity of GP2411 to the reference product has been confirmed on an analytical level and, in a phase I PK and PD study in healthy male subjects, GP2411 demonstrated similarity to Amgen’s Xgeva® (both US-licensed and EU-authorized Xgeva®).

Here, we describe the integrated phase I/phase III ROSALIA study, which aimed to compare the efficacy, PK, PD, immunogenicity, and safety between GP2411 and Amgen’s EU-authorized Prolia® (Amgen Europe B.V. REF-DMAb), including the impact of a treatment switch from REF-DMAb to GP2411, in postmenopausal women with osteoporosis. The results presented herein contribute to the totality of evidence needed to demonstrate similarity between the proposed biosimilar denosumab GP2411 and REF-DMAb.

## Methods

**Figure 1:**
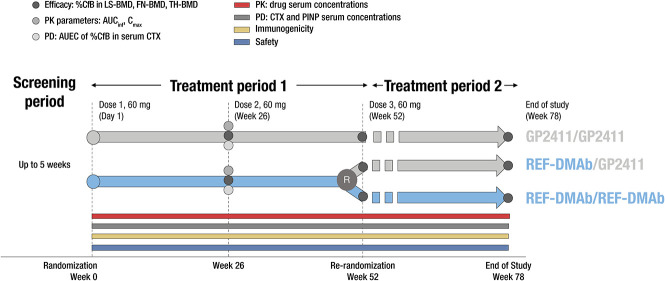
Study design. %CfB, percentage change from baseline; AUC_inf_, area under the serum concentration–time curve extrapolated to infinity; AUEC, area under the effect curve; C_max_, maximum drug serum concentration; PD, pharmacodynamics; PK, pharmacokinetics; REF-DMAb, reference denosumab.

ROSALIA was a multicenter, double-blind, randomized, 2-arm, parallel group, integrated phase I/III study. The study was conducted at 46 sites across Poland (10), Czech Republic (9), USA (9), Bulgaria (7), Spain (6), and Japan (5). In the first treatment period (treatment period 1 [TP1]; weeks 0–52), participants were randomized 1:1 to receive 2 60-mg subcutaneous injections of GP2411 or REF-DMAb (Prolia®), dose 1 at study start (day 1) and dose 2 at week 26. In treatment period 2 [TP2]; weeks 52–78), participants in the REF-DMAb group were re-randomized 1:1 at week 52 to receive dose 3 of REF-DMAb or switch to GP2411; participants received dose 3 at week 52 (Figure 1). Randomization was stratified by region (USA, Japan, rest of the world), age group (<65 yr, ≥65 yr), prior bisphosphonate use (yes, no), and weight category (<70 kg, ≥70 kg). All participants took ≥1000 mg of elemental calcium and 800-IU vitamin D daily from screening until the end of the study. These doses could be reduced or supplemented based on the investigator’s judgement should hypercalcemia or hypocalcemia, respectively, be identified on regular evaluation of calcium levels and serum 25OHD.

The study protocol was approved by the ethics committee or institutional review board for each site. The study was conducted in accordance with the ethical principles of the Declaration of Helsinki, and all participants provided written informed consent. This trial is registered at ClinicalTrials.gov (NCT03974100).

Eligible participants were postmenopausal women with osteoporosis, aged between 55 and 80 yr, with body weight between 50 and 90 kg, and a BMD T-score ≤ −2.5 and ≥ −4.0 at the LS as measured by DXA during screening. Key exclusion criteria included prior exposure to denosumab; ongoing use of osteoporosis treatment (other than calcium and vitamin D supplements); prior use of bisphosphonates (>3 yr of cumulative use prior to screening, and any dose within 12 mo before screening, if oral; and any dose within 5 yr before screening, if intravenous); prior use of glucocorticosteroids within 3 mo before screening; history and/or presence of one severe or more than 2 moderate vertebral fractures; history and/or presence of hip fracture; hypo- or hypercalcemia (hypocalcemia was defined as calcium of <8.42 mg/dL); vitamin D deficiency; current uncontrolled hypo- or hyperthyroidism; or diagnosis (present or historic) of hypo- or hyperparathyroidism; and impaired renal function.

Study endpoints and equivalence criteria were selected to comply with biosimilarity requirements from 3 regulatory authorities (EMA, FDA, and Pharmaceuticals and Medical Devices Agency in Japan [PMDA]). The primary efficacy endpoint was the percent change from baseline (%CfB) in LS-BMD at week 52. The primary PK endpoints were the area under the serum concentration–time curve extrapolated to infinity (AUC_inf_) and maximum drug serum concentration (C_max_) after dose 1 (evaluated at week 26). Additional PK parameters (AUC_last_, AUC_%extrap_, T_max_, Lambda_z, and T1/2) were evaluated and analyzed descriptively after the first dose. The primary PD endpoint was the area under the effect–time curve (AUEC) of the %CfB in serum CTX after dose 1 (evaluated at week 26). Main secondary endpoints included %CfB in LS-BMD, FN-BMD, and TH-BMD at weeks 26 and 78 (and week 52 for FN-BMD and TH-BMD), the %CfB in serum concentration of CTX and P1NP throughout the study, and drug serum concentrations throughout the study.

Immunogenicity was evaluated based on the development of binding antidrug antibodies (ADAs) and neutralizing antibodies (NAbs). Safety endpoints included the incidence of treatment-emergent adverse events (TEAEs), serious TEAEs, and fractures.

Lunar (GE Healthcare, Chicago, IL) and Hologic (Marlborough, MA) DXA scanners were used to determine BMD at the LS and proximal femur. The central imaging vendor Calyx (Billerica, MA) analyzed the DXA scans and provided quality control of the scanners and the individual scans. LS scan included L1 through L4 vertebrae.

Blood samples were collected for PK and PD assessments (in a fasting state for PD) 14 and 12 times, respectively, over 78 wk. Both PK and PD assessments were performed by the Sponsor’s bioanalytical laboratory. Drug serum concentrations were determined by a fully validated enzyme-linked immunosorbent-based method with high sensitivity (lower limit of quantification: 5 ng/mL) and precision (CV ≤11%). CTX and P1NP serum concentrations were determined with by a validated automated immunoassay platform (IDS-iSYS) also comprising high sensitivity (CTX: 0.033 ng/mL; P1NP: 2 ng/mL) and precision (CV ≤15%). Both CTX and P1NP are validated measures of bone turnover, reflective of the effects of osteoporosis treatments, and used for fracture risk prediction. CTX is a reference marker for bone resorption; P1NP is a by-product of the principal constituent of bone (collagen I), a reference marker for bone formation.^12^

A validated immunoassay with a high sensitivity of 9 ng/mL was used. ADAs were assessed based on their titers and neutralizing capacity. Positive (ie, measurable) titer results were defined as ≥20 ng/mL. Participants with persistent ADAs were defined as those with positive results at the last visit and with at least 2 consecutive positive ADA results. Transient ADAs were defined as positive ADA results that were not persistent, ie, ADAs that were detected at single timepoints but with negative results at the following assessment.

Safety was monitored throughout the study. Adverse events were coded with Medical Dictionary for Regulatory Activities, and severity was evaluated with Common Terminology Criteria for Adverse Events v5.0. The central imaging vendor (Calyx) analyzed X-ray images to detect vertebral fractures, defined as an increase in the Genant score from baseline to a later point.

Sample size was estimated for a 2-sided alpha of 5% for efficacy and PD, and 10% for PK, with a total power of at least 90% assuming a 15% dropout rate; assumptions for efficacy were derived from the published trials^13-15^ and for PK and PD from a published model.^16^^,^^17^ Similarity in efficacy was based on the 95% CIs of the least-squares mean treatment difference in %CfB in LS-BMD at week 52 between groups being fully contained within the prespecified equivalence margin of [−1.45%, 1.45%] based on the TP1 full analysis set (FAS) and per-protocol set (PPS). The TP1 FAS consisted of all participants who received at least one dose of study drug and who had at least one post-baseline LS-BMD value. The PPS consisted of participants with LS-BMD assessments at baseline and week 52, who received treatment according to protocol at day 1 and week 26 and who experienced no relevant protocol deviations. Similarity in PK was based on the 90% CIs of the geometric mean ratio for AUC_inf_ and C_max_ being entirely contained within the prespecified equivalence margin of [0.8, 1.25]. Similarity in PD was based on the 95% CI of the geometric mean ratio for the AUEC of %CfB in serum CTX being entirely contained within the prespecified equivalence margin of [0.8, 1.25]. Non-compartmental analysis of PK and PD was performed with Phoenix WinNonlin (Certara, Version 8.3), and all other statistical analyses were conducted with SAS® (SAS Institute, Cary, NC, version 9.4).

## Results

**Figure 2:**
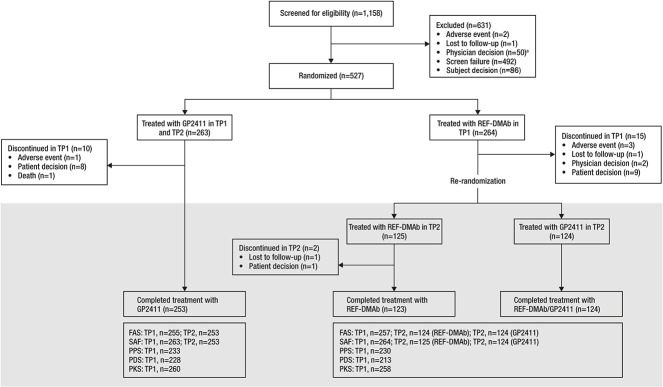
Patient disposition. ^a^Almost all exclusions before randomization because of physician decision (*n* = 49) took place during the COVID-19 pandemic restrictions. COVID-19, coronavirus disease 2019; FAS, full analysis set; PDS, pharmacodynamics analysis set; PKS, pharmacokinetics analysis set; PPS, per-protocol set; REF-DMAb, reference denosumab; SAF, safety analysis set; TP1, treatment period 1; TP2, treatment period 2.

This study was conducted between June 14, 2019 and April 22, 2022. Overall, 527 participants were randomized 1:1 to receive GP2411 (*n* = 263) or REF-DMAb (*n* = 264). Baseline characteristics were similar between the 2 groups; median age was 64.0 yr and 90.9% of participants were White (Table 1). Twenty-five participants discontinued during TP1 (GP2411: *n* = 10; REF-DMAb: *n* = 15), most commonly due to the participants’ decision (*n* = 8 and *n* = 9, respectively). At week 52 (start of TP2), at re-randomization, 124 participants were switched from REF-DMAb to GP2411 (Figure 2). Two participants discontinued during TP2 (both were in the REF-DMAb/REF-DMAb group; one was lost to follow-up and one participant decided to discontinue). Overall, 500 participants completed the study.

**Table 1:** Patient demographics and baseline characteristics (treatment period 1 safety analysis set).

	GP2411 (*n* = 263)	REF-DMAb (*n* = 264)
Age, yr, median	64.0	64.0
Ethnicity, *n* (%)		
Hispanic or Latino	9 (3.4)	8 (3.0)
Not Hispanic or Latino	253 (96.2)	255 (96.6)
Not reported	1 (0.4)	1 (0.4)
Race, *n* (%)		
Asian	23 (8.7)	24 (9.1)
Multiple	1 (0.4)	0
White	239 (90.9)	240 (90.9)
Weight, kg, mean (SD)	62.3 (9.0)	63.4 (9.2)
LS-BMD T-score, mean (SD)	−3.1 (0.42)	−3.1 (0.39)
CTX (ng/mL), median (range)	0.4 (0.0–1.4)	0.4 (0.0–3.4)
P1NP (ng/mL), median (range)	57.6 (10.3–219.6)	56.9 (11.1–256.9)
Prevalent vertebral fractures (≥1), *n* (%)	123 (46.8)	116 (43.9)
Maximum Genant score 1	82 (31.2)	88 (33.3)
Maximum Genant score 2	41 (15.6)	28 (10.6)

Abbreviation: REF-DMAb, reference denosumab.

**Figure 3:**
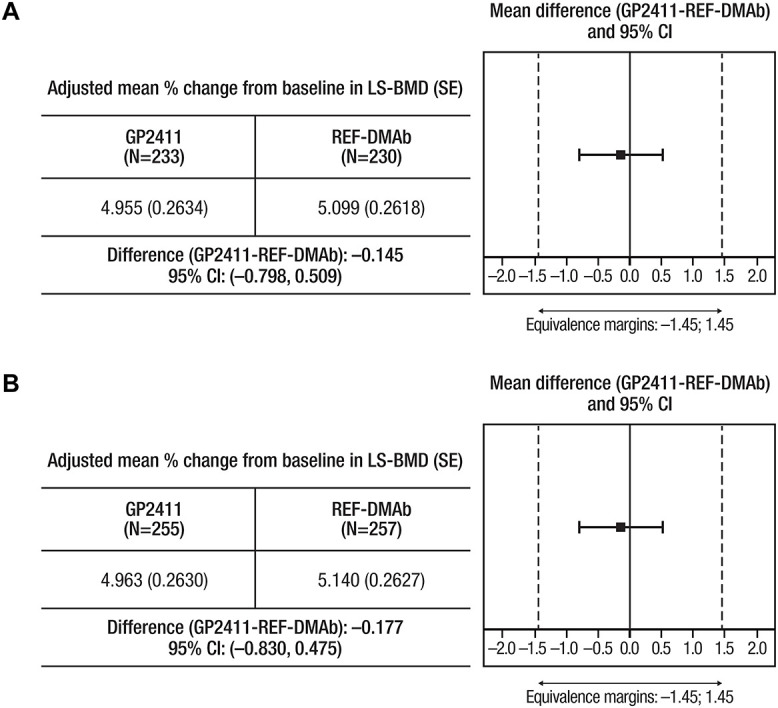
Primary efficacy endpoint. Mean %CfB in LS-BMD and mean difference in %CfB in LS-BMD between GP2411 and REF-DMAb at week 52. (A) Analysis in per-protocol set; (B) analysis in TP1 full-analysis set. Dotted lines mark the equivalence margins. %CfB, percentage change from baseline; REF-DMAb, reference denosumab.

**Figure 4:**
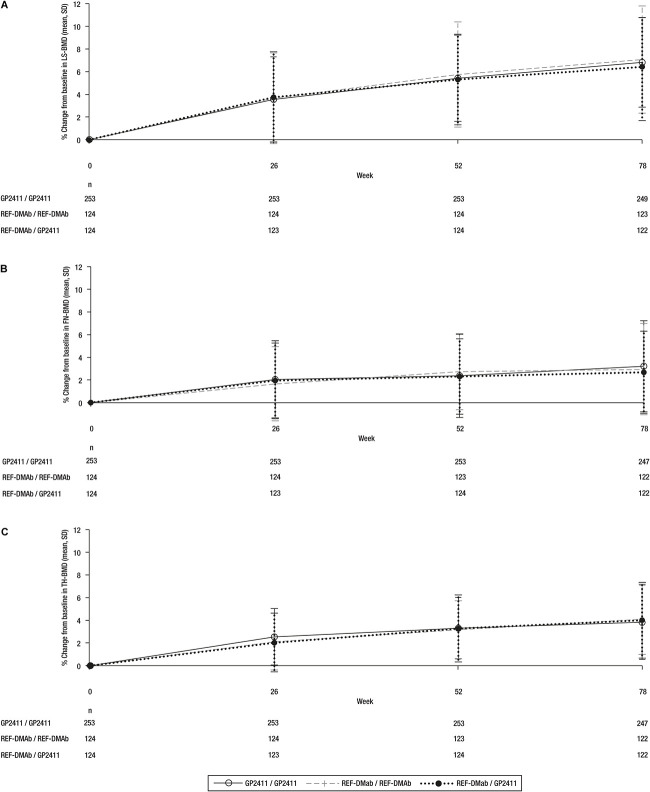
Change in BMD over 78 wk. (A) LS-BMD; (B) FN-BMD; (C) TH-BMD. Data are mean ± SD. REF-DMAb, reference denosumab.

The 95% CIs of the difference in %CfB in LS-BMD between REF-DMAb and GP2411 were fully contained within the prespecified equivalence margins: −0.145 (95% CI: −0.798, 0.509) in the PPS and −0.177 (95% CI: −0.830, 0.475) in the TP1 FAS at week 52 (Figure 3), demonstrating similarity. The %CfB in LS-BMD, FN-BMD, and TH-BMD were also similar between treatment groups in TP1 and TP2 (Supplementary Table S1, Figure 4). The cumulative %CfB (SD) over 78 wk for GP2411/GP2411 was 6.82 (3.95) for LS-BMD, 3.22 (4.04) for FN-BMD, and 3.83 (3.28) for TH-BMD.

**Figure 5:**
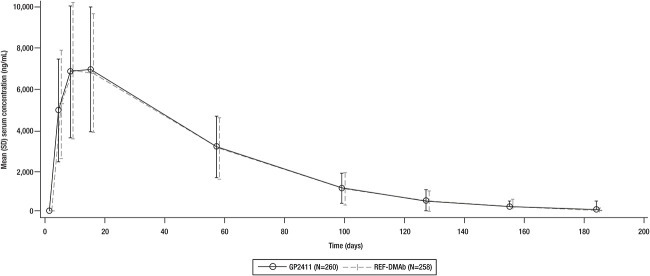
Drug concentrations after the first dose. REF-DMAb, reference denosumab.

The 90% CIs of the geometric mean ratios for AUC_inf_ and C_max_ were fully contained within the prespecified equivalence margins after dose 1 (evaluated at week 26, TP1) (Table 2). Additional PK parameters (AUC_last_, AUC_%extrap_, T_max_, Lambda_z, and T1/2) evaluated after the first dose (evaluated at week 26, TP1) were also similar between treatment groups (Supplementary Table S2). The mean drug serum concentrations were similar between treatment groups up to week 26 (Figure 5) and remained similar throughout the study.

**Figure 6:**
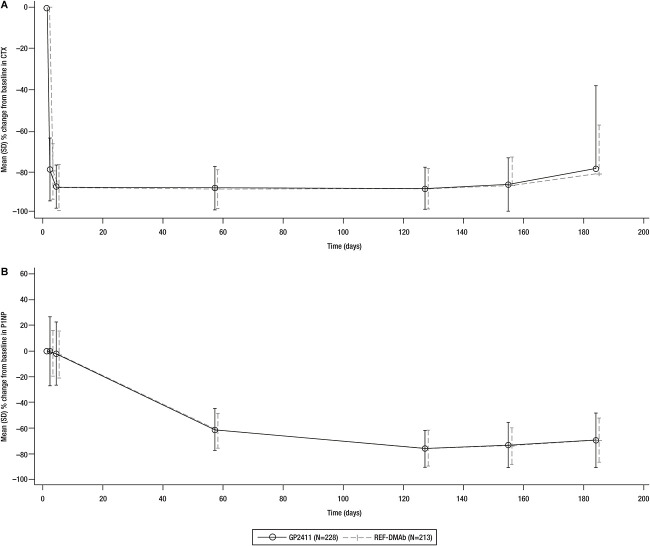
Serum concentration of CTX and P1NP after the first dose. %CfB, percentage change from baseline. REF-DMAb, reference denosumab.

The 95% CIs of the geometric mean ratios of AUEC of %CfB in serum CTX were fully contained within the prespecified equivalence margins after the first dose (evaluated at week 26, TP1). The mean CTX and P1NP serum concentrations and associated %CfB were similar across treatment groups up to week 26 (Figure 6) and remained similar up to and after the treatment switch at week 52.

The 502 participants who received dose 3 at week 52 had a similar incidence of positive ADAs across treatment groups throughout the study (GP2411/GP2411: *n* = 113 [44.7%]; REF-DMAb/REF-DMAb: *n* = 58 [46.4%]; REF-DMAb/GP2411: *n* = 60 [48.4%]), with consistently few positive and low titers (GP2411/GP2411: *n* = 2 [0.8%]; REF-DMAb/REF-DMAb: *n* = 2 [1.6%]; REF-DMAb/GP2411: *n* = 0). The vast majority (98% overall) of all participants with positive ADAs had ADAs that were borderline detectable by the highly sensitive method, which resulted in non-measurable titers (ie, titer negative results). Overall, only 13 participants (with similar distribution across groups) had a measurable persistent ADA response (GP2411/GP2411: *n* = 5 [2.0%]; REF-DMAb/REF-DMAb: *n* = 5 [4.0%]; REF-DMAb/GP2411: *n* = 3 [2.4%]). At any time during the study, the incidence of NAbs was very low and similar across treatment groups (0.8% in all 3 treatment groups). Overall, detected immunogenicity was not clinically meaningful based on the very low magnitude of ADAs and NAbs in terms of titer. Furthermore, switching from REF-DMAb to GP2411 did not affect immunogenicity compared with the non-switched population.

The rate of any-grade TEAEs was similar between treatment groups in TP1 (GP2411: *n* = 157 [59.7%]; REF-DMAb: *n* = 181 [68.6%]) and TP2 (GP2411/GP2411: *n* = 68 [26.9%]; REF-DMAb/REF-DMAb: *n* = 47 [37.6%]; REF-DMAb/GP2411: *n* = 48 [38.7%]) (Supplementary Table S3). Most TEAEs during TP1 and TP2 were of grade 1 or 2. The rate of serious TEAEs was similar between treatment groups in TP1 (GP2411: *n* = 12 participants [4.6%]; REF-DMAb: *n* = 8 [3.0%]) and TP2 (GP2411/GP2411: *n* = 4 [1.6%]; REF-DMAb/REF-DMAb: *n* = 2 [1.6%]; REF-DMAb/GP2411: *n* = 0).

In TP1, TEAEs led to discontinuation of study drug in 5 participants in TP1 (GP2411: *n* = 1 [0.4%]; REF-DMAb: *n* = 4 [1.5%]) and to study discontinuation in 6 participants (GP2411: *n* = 3 [1.1%]; REF-DMAb: *n* = 3 [1.1%]). No TEAEs led to discontinuation of study in TP2.

The cumulative incidences of all-grade TEAEs (GP2411/GP2411: *n* = 161 [63.6%]; REF-DMAb/REF-DMAb: *n* = 95 [76.0%]; REF-DMAb/GP2411: *n* = 95 [76.6%]), and their severity, were comparable between treatment groups (Supplementary Table S4). Throughout the study, most TEAEs were of grade 1 or 2.

The most frequent TEAE was hypocalcemia (GP2411/GP2411: *n* = 27 [10.7%]; REF-DMAb/REF-DMAb: *n* = 14 [11.2%]; REF-DMAb/GP2411: *n* = 12 [9.7%]). Hypocalcemia was most frequently reported at visits 5 and 6 (1 and 2 wk after the first injection, respectively). In TP1 overall, hypocalcemia affected 28 patients (10.6%) for GP2411 and 26 patients (9.8%) for REF-DMAb and, in TP2, it affected one patient only (0.4%) who received GP2411/GP2411. All cases were grade 1 or 2. Other common TEAEs were nasopharyngitis (GP2411/GP2411: *n* = 25 [9.9%]; REF-DMAb/REF-DMAb: *n* = 8 [6.4%]; REF-DMAb/GP2411: *n* = 16 [12.9%]) and coronavirus disease 2019 (COVID-19) (GP2411/GP2411: *n* = 15 [5.9%]; REF-DMAb/REF-DMAb: *n* = 13 [10.4%]; REF-DMAb/GP2411: *n* = 8 [6.5%]) (Supplementary Table S4).

There were no clinically meaningful differences between groups in the overall incidence of new vertebral fractures (GP2411/GP2411: *n* = 25 participants [9.9%]; REF-DMAb/REF-DMAb: *n* = 17 participants [13.6%]; REF-DMAb/GP2411: *n* = 17 participants [13.7%]), or their severity, throughout the study. This was also true for non-hip non-vertebral fractures at 52 wk (*n* = 2 participants [0.8% in both arms]) and 78 wk (GP2411/GP2411: *n* = 1 participants [0.4%]; REF-DMAb/REF-DMAb: *n* = 0 participants; REF-DMAb/GP2411: *n* = 1 participants [0.8%]).

Overall, switching from REF-DMAb to GP2411 did not result in increased adverse events indicative of hypersensitivity reactions. There was one death in this study: a participant receiving GP2411 who died of unknown cause in TP1. The participant was elderly with preexisting cardiovascular comorbidities, and their death was deemed unrelated to the study drug.

**Table 2:** PK and PD evaluation after the first treatment dose.

	Adjusted geometric mean		Geometric mean ratio GP2411/REF-DMAb	
	GP2411	REF-DMAb	PE	(90% CI)
AUC_inf_ (day * ng/mL)	366 000 *n* = 247	369 000 *n* = 246	0.99	(0.93, 1.05)
C_max_ (ng/mL)	6910 *n* = 260	7120 *n* = 258	0.97	(0.92, 1.03)
	**GP2411**	**REF-DMAb**	**PE**	**(95% CI)**
AUEC %CfB in CTX (% * day)	15 800 *n* = 228	15 800 *n* = 213	1.00	(0.98, 1.01)

Abbreviations: AUC_inf_, area under the serum concentration–time curve extrapolated to infinity; AUEC, area under the effect curve; C_max_, maximum drug serum concentration; PD, pharmacodynamics; PE, point estimate; PK, pharmacokinetics; *n*, number of participants with evaluable parameters; REF-DMAb, reference denosumab.

## Discussion

This study demonstrated similarity in efficacy, PK, PD, immunogenicity, and safety between proposed biosimilar denosumab, GP2411, and EU-authorized REF-DMAb in postmenopausal women with osteoporosis. Efficacy was similar between GP2411 and REF-DMAb in terms of the effect of treatment on BMD. PK and PD were similar between GP2411 and REF-DMAb, with CIs for all endpoints contained within the prespecified equivalence margins. Additionally, serum concentrations of study drugs and of bone turnover markers CTX and P1NP were also similar between treatment groups throughout the study. GP2411 was well tolerated, with most TEAEs of grade 1–2, similar rates of TEAEs and serious AEs across treatment groups, and a similar effect to REF-DMAb in the rate of new vertebral fractures. Safety findings were generally in line with the known profile of denosumab (eg, hypocalcemia)^2^ The incidence of hypocalcemia was slightly higher than in the pivotal phase III FREEDOM trial of denosumab vs placebo in postmenopausal women with osteoporosis,^13^ and this may reflect the higher threshold for a diagnosis of hypocalcemia (<8.42mg/dL vs <8.0 mg/dL). No new safety findings were identified. In addition to meeting the study’s main objectives, our results indicate that GP2411 had no clinically meaningful differences in efficacy, and no impact on immunogenicity or safety, in patients who underwent a single switch from REF-DMAb to GP2411.

In this study, we demonstrated similar immunogenicity between treatment groups—GP2411 and REF-DMAb—in terms of incidence, magnitude (titer), and neutralizing capacity of ADAs. The rates identified here (ranging from 44.7% to 48.4% of participants) are higher than those reported in previous denosumab studies (<1%). Indeed, they were not reported in any participant in the pivotal phase III FREEDOM trial of denosumab vs placebo in postmenopausal women with osteoporosis.^1^^,^^2^^,^^13^ This is likely a result of the high sensitivity (9 ng/mL) of the ADA assay used in the current study, which was to fulfil regulatory requirements of biosimilar evaluation.^18^ ADAs with concentrations of 100 ng/mL or higher may be associated with clinical events^19^; the method used here to detect ADAs (with a sensitivity of 9 ng/mL) is, therefore, approximately 10 times more sensitive than that required to detect clinically meaningful immunogenicity. Moreover, for >98% of participants with detected ADAs, these were borderline detectable by the method and consequently reported as “ADA titer-negative,” which demonstrates the low immunogenic capacity of both GP2411 and REF-DMAb in this study. This is further supported by the overall non-neutralizing and transient nature of the vast majority of observed ADAs in this study. Titers above the cut-point of 20 ng/mL could not be reported using a mass/volume unit, as was performed in this study, because the person-specific nature of ADAs prevents a calibration curve from being created.

The FREEDOM trial established the role of REF-DMAb in increasing BMD, reducing bone turnover, and decreasing the risk of vertebral, hip, and non-vertebral fracture rates vs placebo in postmenopausal women with osteoporosis. ^(^^13^^,^^20^^)^ The %CfB for LS-BMD, FN-BMD, and TH-BMD reported over 78 wk in this study are similar to those reported during the initial 1–2 yr of the FREEDOM trial, representing a similar time period.^20^

Regulatory authorities differ in their requirements for demonstrating biosimilarity.^21^ In this study, efficacy, PK, and PD endpoints were selected in alignment with various regulatory authorities (FDA, EMA, and PMDA), and equivalence was demonstrated across all endpoints. The bone turnover markers that were used (CTX for bone resorption and P1NP for bone formation) are those recommended as reference markers by the International Osteoporosis Foundation and the International Federation of Clinical Chemistry and Laboratory Medicine.^12^ Another strength of this study was the large sample size, the stratified randomization, and the evaluation of GP2411 in patients who underwent a single switch from REF-DMAb. The treatment duration of 52 wk matched the median treatment duration reported for 29 other comparative biosimilar studies,^22^ demonstrating a suitable time period for evaluating equivalence. The limitations include the use of a primarily White, non-Hispanic population and fractures not being the primary outcome. In addition, the ROSALIA study was initiated prior to and completed during the COVID-19 pandemic. The potential impact of this on conduct and outcomes was assessed throughout the study. Although the pandemic caused a recruitment pause at the beginning of the pandemic, few missed or delayed visits, samples, measurements, or on-site monitoring visits, it was not considered to have affected the study population, integrity, or validity of the study results.

In summary, this phase I/III study demonstrated similarity between GP2411 and REF-DMAb in terms of efficacy, PK, PD, immunogenicity, and safety. These findings contribute to the overall totality of evidence supporting the biosimilarity of GP2411 and REF-DMAb.

## Supplementary Material

231106_Sandoz_ROSALIA_Supplement_v5_0_Submitted_zjae016

## Data Availability

The full protocol and these data are available on request from the corresponding author. This trial is also registered at ClinicalTrials.gov (NCT03974100).
