# Correction to: Equivalence trial of proposed denosumab biosimilar GP2411 and reference denosumab in postmenopausal osteoporosis: the ROSALIA study

**DOI:** 10.1093/jbmr/zjae077

**Published:** 2024-05-22

This is a correction to: Slawomir Jeka, Eva Dokoupilová, Alan Kivitz, Pawel Zuchowski, Barbara Vogg, Natalia Krivtsova, Susmit Sekhar, Samik Banerjee, Arnd Schwebig, Johann Poetzl, Jean-Jacques Body, Richard Eastell, Equivalence trial of proposed denosumab biosimilar GP2411 and reference denosumab in postmenopausal osteoporosis: the ROSALIA study, *Journal of Bone and Mineral Research*, Volume 39, Issue 3, March 2024, Pages 202–210, https://doi.org/10.1093/jbmr/zjae016.

In the originally published version of this manuscript, an error was identified in the results text, arising from column-row confusion. The following sentence was corrected from: The cumulative %CfB (SD) over 78 wk was 6.82 (3.95) for LS-BMD, 7.07 (4.73) for FN-BMD, and 6.42 (4.47) for TH-BMD' to: ‘The cumulative %CfB (SD) over 78 wk for GP2411/GP2411 was 6.82 (3.95) for LS-BMD, 3.22 (4.04) for FN-BMD, and 3.83 (3.28) for TH-BMD.’ The relevant values were and are also included, correctly, in the supplementary data.

Minor amends were also made to display items, with no change to the overall findings: ‘Subject decision (n = 86)’ was added to the list of screening exclusions in Figure 2; the baseline n-number was amended to ‘253’ in Figure 4; and ‘REF-DMAb/GP2411’ was changed to ‘GP2411/REF-DMAb’ in the column heading of Table 2.

