# Supplementary material for: Equivalence trial of proposed denosumab biosimilar GP2411 and reference denosumab in postmenopausal osteoporosis: the ROSALIA study
Source: J Bone Miner Res. 2024 Jan 29;39(3):202–10. doi: 10.1093/jbmr/zjae016 (PMC11338045; doi:10.1093/jbmr/zjae016)
Supplement: 231106_Sandoz_ROSALIA_Supplement_v5_0_Submitted_zjae016 [file 231106_Sandoz_ROSALIA_Supplement_v5_0_Submitted_zjae016.docx]

**SUPPLEMENTARY MATERIAL**

Supplementary Table 1. Efficacy data (full analysis set).

|  | **Treatment Period 1** | | **Treatment Period 1 + Treatment Period 2** | | |
| --- | --- | --- | --- | --- | --- |
| **Efficacy parameter,**  **mean (SD), n** | **GP2411** | **REF-DMAb** | **GP2411/ GP2411** | **REF-DMAb/REF-DMAb** | **REF-DMAb/GP2411** |
|  | **Week 26** | | | | |
| %CfB LS-BMD | 3.59 (3.74), n=255 | 3.71 (3.90), n=256 | 3.56 (3.75), n=253 | 3.67 (3.94), n=124 | 3.75 (3.94), n=123 |
| %CfB FN-BMD | 2.03 (3.44), n=254 | 1.82 (3.11), n=256 | 2.03 (3.45), n=252 | 1.66 (3.16), n=124 | 1.94 (3.12), n=123 |
| %CfB TH-BMD | 2.53 (2.47), n=254 | 2.06 (2.52), n=256 | 2.54 (2.48), n=252 | 2.10 (2.38), n=124 | 2.01 (2.55), n=123 |
|  | **Week 52** | | | | |
| %CfB LS-BMD | 5.45 (3.79), n=253 | 5.61 (4.20), n=249 | 5.42 (3.80), n=253 | 5.75 (4.52), n=124 | 5.31 (3.90), n=124 |
| %CfB FN-BMD | 2.37 (3.69), n=253 | 2.57 (3.30), n=248 | 2.37 (3.69), n=253 | 2.75 (3.29), n=123 | 2.33 (3.22), n=124 |
| %CfB TH-BMD | 3.29 (2.70), n=253 | 3.22 (2.65), n=248 | 3.30 (2.71), n=253 | 3.17 (2.42), n=123 | 3.26 (2.87), n=124 |
|  | **Week 78** | | | | |
| %CfB LS-BMD | — | — | 6.82 (3.95), n=249 | 7.07 (4.73), n=123 | 6.42 (4.47), n=122 |
| %CfB FN-BMD | — | — | 3.22 (4.04), n=247 | 2.94 (3.92), n=122 | 2.69 (3.64), n=122 |
| %CfB TH-BMD | — | — | 3.83 (3.28), n=247 | 4.09 (2.97), n=122 | 4.00 (3.33), n=122 |

%CfB, percentage change from baseline; FN-BMD, femoral neck bone mineral density; LS-BMD, lumbar spine bone mineral density, REF-DMAb, reference denosumab; SD, standard deviation; TH-BMD, total hip bone mineral density.

Supplementary Table 2. Additional PK outcomes in treatment period 1 (Day 1 to Week 52).

| **Parameter, mean (SD)** | **GP2411** | **REF-DMAb** |
| --- | --- | --- |
| AUC_last_ (day* ng/mL) | 394,000 (173000)  (n=260) | 385,000 (176000)  (n=258) |
| AUC_extrap_ (%) | 1.020 (1.90)  (n=247) | 0.850 (1.99)  (n=246) |
| Lambda_z (1/day) | 0.045 (0.017)  (n=247) | 0.045 (0.016)  (n=246) |
| T1/2 (day) | 18.2 (8.49)  (n=247) | 17.7 (8.06)  (n=246) |
| T_max_ (day), median | 13.0  (n=260) | 9.94  (n=258) |

AUC_extrap_, percentage of AUC_inf_ due to extrapolation from the time of the last observed concentration to infinity; AUC_inf_, area under the serum concentration–time curve measured from the time of dosing and extrapolated to infinity; AUC_last_, area under the concentration–time curve from dosing to the time of the last measured concentration; n, number of participants with evaluable parameters; PK, pharmacokinetics; REF-DMAb, reference denosumab; SD, standard deviation.

Supplementary Table 3. Safety summary.

|  | **Treatment Period 1** | | | | **Treatment Period 2** | | | | | |
| --- | --- | --- | --- | --- | --- | --- | --- | --- | --- | --- |
|  | **GP2411 (n=263)** | | **REF-DMAb**  **(n=264)** | | **GP2411/GP2411**  **(n=253)** | | **REF-DMAb/ REF-DMAb**  **(n=125)** | | **REF-DMAb/ GP2411**  **(n=124)** | |
| n, (%) | All grades | Grade 3–4 | All grades | Grade 3–4 | All grades | Grade 3–4 | All grades | Grade 3–4 | All grades | Grade 3–4 |
| At least one TEAE | 157 (59.7) | 13  (4.9) | 181 (68.6) | 10  (3.8) | 68  (26.9) | 5  (2.0) | 47  (37.6) | 2  (1.6) | 48  (38.7) | 2  (1.6) |
| Treatment-related | 36 (13.7) | 0 | 49  (18.6) | 0 | 7  (2.8) | 0 | 7  (5.6) | 0 | 5  (4.0) | 0 |
| At least one serious TEAE | 12  (4.6) | 9  (3.4) | 8  (3.0) | 5  (1.9) | 4  (1.6) | 3  (1.2) | 2  (1.6) | 1  (0.8) | 0 | 0 |
| Treatment-related | 0 | 0 | 0 | 0 | 0 | 0 | 0 | 0 | 0 | 0 |
| Fatal serious TEAE | 1  (0.4)^a^ | 0 | 0 | 0 | 0 | 0 | 0 | 0 | 0 | 0 |
| Treatment-related | 0 | 0 | 0 | 0 | 0 | 0 | 0 | 0 | 0 | 0 |
| TEAE leading to study drug discontinuation | 1  (0.4) | 1  (0.4) | 4  (1.5) | 3  (1.1) | 0 | 0 | 0 | 0 | 0 | 0 |
| Treatment-related | 0 | 0 | 0 | 0 | 0 | 0 | 0 | 0 | 0 | 0 |
| TEAE leading to study discontinuation | 3  (1.1) | 1  (0.4) | 3  (1.1) | 2  (0.8) | 0 | 0 | 0 | 0 | 0 | 0 |
| Treatment-related | 0 | 0 | 0 | 0 | 0 | 0 | 0 | 0 | 0 | 0 |

n, number of participants; REF-DMAb, reference denosumab; TEAE, treatment-emergent adverse event.

^a^The participant was elderly with pre-existing cardiovascular comorbidities, and died of unknown causes considered not to be related to the study drug. Fatal TEAEs (death) were classified as Grade 5.

Supplementary Table 4. Summary of cumulative adverse events by system organ class (reported in ≥5% of patients in any treatment group).

|  | **GP2411/GP2411**  **(n=253)** | | **REF-DMAb/REF/DMAb**  **(n=125)** | | **REF-DMAb/GP2411**  **(n=124)** | |
| --- | --- | --- | --- | --- | --- | --- |
| Primary system organ class Preferred term  n, (%) | All Grades | Grade 3–4 | All Grades | Grade 3–4 | All Grades | Grade 3–4 |
| Number of participants with at least one TEAE | 161 (63.6) | 14 (5.5) | 95 (76.0) | 6 (4.8) | 95 (76.6) | 4 (3.2) |
| Infections and infestations | 74 (29.2) | 2 (0.8) | 50 (40.0) | 2 (1.6) | 45 (36.3) | 2 (1.6) |
| Nasopharyngitis | 25 (9.9) | 0 | 8 (6.4) | 0 | 16 (12.9) | 0 |
| COVID-19 | 15 (5.9) | 2 (0.8) | 13 (10.4) | 0 | 8 (6.5) | 1 (0.8) |
| Urinary tract infection | 6 (2.4) | 0 | 7 (5.6) | 0 | 4 (3.2) | 0 |
| Musculoskeletal and connective tissue disorders | 56 (22.1) | 1 (0.4) | 31 (24.8) | 1 (0.8) | 27 (21.8) | 1 (0.8) |
| Arthralgia | 15 (5.9) | 0 | 7 (5.6) | 0 | 5 (4.0) | 0 |
| Back pain | 11 (4.3) | 1 (0.4) | 7 (5.6) | 0 | 4 (3.2) | 1 (0.8) |
| Metabolism and nutrition disorders | 45 (17.8) | 0 | 22 (17.6) | 0 | 26 (21.0) | 0 |
| Hypocalcemia | 27 (10.7) | 0 | 14 (11.2) | 0 | 12 (9.7) | 0 |
| Vitamin D deficiency | 8 (3.2) | 0 | 8 (6.4) | 0 | 9 (7.3) | 0 |
| Gastrointestinal disorders | 38 (15.0) | 3 (1.2) | 24 (19.2) | 1 (0.8) | 18 (14.5) | 0 |
| Diarrhea | 4 (1.6) | 0 | 7 (5.6) | 0 | 3 (2.4) | 0 |
| General disorders and administration site conditions | 25 (9.9) | 0 | 9 (7.2) | 0 | 15 (12.1) | 0 |
| Injection site reaction | 8 (3.2) | 0 | 2 (1.6) | 0 | 8 (6.5) | 0 |
| Injury, poisoning and procedural complications | 25 (9.9) | 6 (2.4) | 12 (9.6) | 0 | 17 (13.7) | 1 (0.8) |
| Contusion | 4 (1.6) | 0 | 4 (3.2) | 0 | 7 (5.6) | 0 |
| Nervous system disorders | 17 (6.7) | 0 | 14 (11.2) | 0 | 13 (10.5) | 1 (0.8) |
| Headache | 5 (2.0) | 0 | 3 (2.4) | 0 | 9 (7.3) | 1 (0.8) |
| Skin and subcutaneous tissue disorders | 12 (4.7) | 1 (0.4) | 10 (8.0) | 0 | 6 (4.8) | 0 |

REF-DMAb, reference denosumab; TEAE, treatment-emergent adverse event.
